# A comparison of effects of DPP-4 inhibitor and SGLT2 inhibitor on lipid profile in patients with type 2 diabetes

**DOI:** 10.1186/s12944-017-0443-4

**Published:** 2017-04-13

**Authors:** Seon-Ah Cha, Yong-Moon Park, Jae-Seung Yun, Tae-Seok Lim, Ki-Ho Song, Ki-Dong Yoo, Yu-Bae Ahn, Seung-Hyun Ko

**Affiliations:** 1 Department of Internal Medicine, Division of Endocrinology and Metabolism, College of Medicine, The Catholic University of Korea, St. Vincent’s Hospital, 93 Jungbu − daero, Paldal − gu, Suwon, Gyeonggi − do, Seoul, 442-723 Republic of Korea; 2 Epidemiology Branch, National Institute of Environmental Health Sciences, National Institutes of Health, Research Triangle Park, Durham, NC USA; 3 Department of Internal Medicine, Division of Cardiology, College of Medicine, The Catholic University of Korea, Seoul, Republic of Korea

**Keywords:** DPP-4 inhibitor, SGLT2 inhibitor, Lipid, Type 2 diabetes

## Abstract

**Background:**

Previous studies suggest that dipeptidyl peptidase-4 (DPP-4) inhibitors and sodium glucose cotransporter 2 (SGLT2) inhibitors have different effects on the lipid profile in patients with type 2 diabetes. We investigated the effects of DPP-4 inhibitors and SGLT2 inhibitors on the lipid profile in patients with type 2 diabetes.

**Methods:**

From January 2013 to December 2015, a total of 228 patients with type 2 diabetes who were receiving a DPP-4 inhibitor or SGLT2 inhibitor as add-on therapy to metformin and/or a sulfonylurea were consecutively enrolled. We compared the effects of DPP-4 inhibitors and SGLT2 inhibitors on the lipid profile at baseline and after 24 weeks of treatment. To compare lipid parameters between the two groups, we used the analysis of covariance (ANCOVA).

**Results:**

A total of 184 patients completed follow-up (mean age: 53.1 ± 6.9 years, mean duration of diabetes: 7.1 ± 5.7 years). From baseline to 24 weeks, HDL-cholesterol (HDL-C) levels were increased by 0.5 (95% CI, −0.9 to 2.0) mg/dl with a DPP-4 inhibitor and by 5.1 (95% CI, 3.0 to 7.1) mg/dl with an SGLT2 inhibitor (*p* = 0.001). LDL-cholesterol (LDL-C) levels were reduced by 8.4 (95% CI, −14.0 to -2.8) mg/dl with a DPP-4 inhibitor, but increased by 1.3 (95% CI, −5.1 to 7.6) mg/dl with an SGLT2 inhibitor (*p* = 0.046). There was no significant difference in the mean hemoglobin A1c (8.3 ± 1.1 vs. 8.0 ± 0.9%, *p* = 0.110) and in the change of total cholesterol (TC) (*p* = 0.836), triglyceride (TG) (*p* = 0.867), apolipoprotein A (*p* = 0.726), apolipoprotein B (*p* = 0.660), and lipoprotein (a) (*p* = 0.991) between the DPP-4 inhibitor and the SGLT2 inhibitor.

**Conclusions:**

The SGLT2 inhibitor was associated with a significant increase in HDL-C and LDL-C after 24 weeks of SGLT2 inhibitor treatment in patients with type 2 diabetes compared with those with DPP-4 inhibitor treatment in this study.

**Trial registration:**

This study was conducted by retrospective medical record review.

**Electronic supplementary material:**

The online version of this article (doi:10.1186/s12944-017-0443-4) contains supplementary material, which is available to authorized users.

## Background

Diabetes mellitus is related to an increased risk of cardiovascular disease (CVD) [1]. In Korea, a risk of coronary heart disease and stroke were 4 times and 2 times higher in patients with diabetes compared with those without diabetes, respectively [2]. CVD is the major cause of morbidity and cardiovascular mortality in patients with type 2 diabetes [3–5]. Diabetes with CVD has average annual per-person medical care costs adjusted for age and sex that are 1.6-fold higher than those without diabetes [6].

Contributing factors that increase the risk of CVD include hypertension, dyslipidemia, obesity, and smoking in patients with diabetes [4]. Dyslipidemia is common in patients with type 2 diabetes, which is characterized by low HDL-cholesterol (HDL-C), elevated triglycerides (TG), and a predominance of small, dense LDL particles [7, 8].

The American Diabetes Association (ADA) and American College of Cardiology Foundation recommend that lifestyle intervention and pharmacologic therapy be started concurrently in patients with type 2 diabetes, regardless of LDL-cholesterol (LDL-C) [9]. In its recent guideline, the ADA recommended pharmacologic therapy, primarily statin therapy, in patients with type 2 diabetes who have any CVD risk factors or patients 40 years of age or older [10].

Despite the evidence that lowered LDL-C could lead to reduced risk of CVD, it is estimated that nearly half of patients with type 2 diabetes did not achieve current LDL-C goals [11, 12]. Thus, a relatively large number of patients with type 2 diabetes are exposed to the risks of CVD [13].

A dipeptidyl peptidase-4 (DPP-4) inhibitor is an oral hypoglycemic agent that exerts its effect by inactivating incretin, which is released from the intestinal cells after meal ingestion [11]. In Korea, the use of DPP-4 inhibitors has increased in the last decade, and DPP-4 inhibitors comprised one-third of the market share in 2013 [14]. Previous studies reported that DPP-4 inhibitors have effects on total cholesterol (TC), but results are variable across trials. A recent meta-analysis reported a possible beneficial effect of DPP-4 inhibitors including vildagliptin and alogliptin on TC and TG levels compared to placebo [15].

A sodium glucose cotransporter 2 (SGLT2) inhibitor is an antihyperglycemic agent that effectively improves glycemic control through inhibiting glucose absorption in the proximal tubule of the kidney [16]. In addition to improving glycemic control, SGLT2 inhibitors are reported to have additional beneficial effects on body weight and blood pressure, with a low risk of hypoglycemia. SGLT2 inhibitors are also reported to have an association with increases in HDL-C and LDL-C [17]. The mechanism that an SGLT2 inhibitor increases LDL-C levels remains unknown, and a dose-related increase in LDL-C has been observed in patients who were given an SGLT2 inhibitor [18].

DPP-4 inhibitors and SGLT2 inhibitors are both a treatment option as monotherapy or as part of dual and triple therapy in patients with type 2 diabetes, having different effects on the lipid profile. This study compared the effects of two DPP-4 inhibitors (linagliptin, gemigliptin) and an SGLT2 inhibitor (dapagliflozin) on the lipid profile in patients with type 2 diabetes.

## Methods

Through retrospective medical record review, a total of 228 patients with type 2 diabetes, aged 25–65 years and who did not achieve glycemic goals with metformin and/or a sulfonylurea and were receiving linagliptin or gemigliptin or dapagliflozin as add-on therapy, were consecutively recruited from January 2013 to December 2015, and scheduled for follow-up from July 2013 to June 2016 at the university-affiliated diabetes center of St. Vincent’s Hospital in South Korea.

Patients were excluded from this study for any of the following: type 1 diabetes, any change in previous medications that could influence their lipid profile including statins, fibric acid agents, niacin, omega acid ethyl esters, thyroid hormones, steroids within 3 months before enrollment or during the period of DPP-4 inhibitor or SGLT2 inhibitor treatment, fasting serum TG ≥ 600 mg/dl at screening, or estimated glomerular filtration rate (eGFR) less than 60 ml/min/1.73 m^2^. The enrolled patients were divided into two groups (DPP-4 inhibitor group or SGLT2 inhibitor group) according to the drugs that they were receiving. This study was conducted in accordance with the Declaration of Helsinki guidelines, and it was approved by investigator’s institutional ethical review board.

Information including participant’s history, ex- or current cigarette smoking status, and use of medication were collected at the beginning of the study. Hypertension was defined as systolic blood pressure of 140 mmHg or greater, diastolic blood pressure of 90 mmHg or greater, or current use of antihypertensive medications.

The fasting blood samples, including the levels of fasting plasma glucose (FPG), hemoglobin A1c (HbA1c), TC, TG, HDL-C, LDL-C, lipoprotein (a) (Lp[a]), apolipoprotein A, apolipoprotein B and creatinine, aspartate aminotransferase, and alanine transaminase, were measured at baseline and 24 weeks after DPP-4 inhibitor or SGLT2 inhibitor therapy.

The FPG and lipid profile were assessed using an automated enzymatic method (736–40; Hitachi, Tokyo, Japan), and HbA1c was assessed using high-performance liquid chromatography (Bio-Rad, Montreal, QC, Canada). Serum Lp(a) concentration was measured using a one-step sandwich enzyme-linked immunoassay (TintElize Lp(a) kt, Biopool AB, Umea, Sweden) [19]. The eGFR was assessed using the four-component Modification of Diet in Renal Disease equation [20]. The urinary albumin excretion rates were measured from single-void urine specimens using immunoturbidimetry (Eiken, Tokyo, Japan). C-peptide and insulin levels were measured using a chemiluminescent microparticle immunoassay (ARCHITECT C-Peptide Reagent Kit [CL53], Biokit S.A., Barcelona, Spain, ARCHITECT Insulin, Denka Seiken Co., Ltd. Tokyo, Japan). Insulin resistance and insulin secretion were estimated by homeostatic model assessment-insulin resistance (HOMA-IR) and the HOMA β-Cell function.

Diabetic retinopathy was assessed through a comprehensive eye examination by an ophthalmologist from retinal photographs taken at baseline. Diabetic nephropathy was defined as a urine albumin-to-creatinine ratio > 30 mg/g of creatinine in spot urine specimens [21].

### Statistical analysis

All data are expressed as the mean ± standard deviation or frequencies or medians with an interquartile range or 95% confidence interval. The categorical variables were tested using Chi-square test, and independent Student’s t-tests evaluated the differences between the means of two continuous variables. The Mann–Whitney U test was used for non-normally distributed variables.

Differences in body weight, blood pressure, FPG, HbA1c, and serum lipids between baseline and after 24 weeks of treatment of a DPP-4 inhibitor and an SGLT2 inhibitor were analyzed by the paired *t*-test. Changes in TG and HDL-C were assessed by Wilcoxon signed-rank test.

We determined the effect of a DPP-4 inhibitor and an SGLT2 inhibitor on the lipid profile between baseline and 24 weeks, using analysis of covariance (ANCOVA) with treatment as the factor and using age, sex, diabetes duration, body mass index (BMI), and change of HbA1c (%) as covariates.

The assessment of side effects, including the incidence of adverse events, was described without tests for significance. Statistical analyses were performed using SAS version 9.3 (SAS Institute, Cary, NC, USA). *p* < 0.05 was considered significant.

## Results

Of 228 patients who were recruited, 184 patients (80.7%) completed follow-up. One patient who received insulin and 26 patients who underwent any change in medication that can influence lipid profile during the follow-up period, and five patients who had an eGFR less than 60 ml/min/1.73 m^2^ at baseline, were excluded. The planned follow-up period was 6 months.

Among 184 patients, 55 patients had taken linagliptin, 69 patients had taken gemigliptin, and 60 patients had taken dapagliflozin. Baseline demographic, anthropometric, laboratory finding, and treatment characteristics of subjects are summarized in Table 1.

**Table 1:** Comparison of baseline characteristics between the subjects with DPP-4 inhibitor and SGLT2 inhibitor

	Total	DPP-4 inhibitors Linagliptin (*n =* 55) Gemigliptin (*n =* 69)	SGLT2 inhibitor	*p*-value
n	184	124	60	
Women (n, %)	97 (52.7)	62 (50.0)	35 (58.3)	0.289
Age (years)	53.1 ± 6.9	53.4 ± 7.1	52.6 ± 6.5	0.474
Duration of diabetes (years)	6.0 (3.0–11.0)	5.0 (2.0–10.8)	7.0 (3.0–11.0)	0.586
Hypertension (n, %)	75 (41.0)	47 (37.9)	28 (46.7)	0.257
Smoking (n, %)				0.150
Current (n, %)	36 (22.6)	30 (25.9)	6 (12.5)	
Ex-smoker (n, %)	18 (10.1)	13 (11.2)	5 (10.4)	
Body weight (kg)	68.0 (62.0–77.0)	67.0 (62.0–75.0)	70.0 (62.0–80.8)	0.326
BMI (kg/m^2^)	26.0 ± 3.4	25.6 ± 3.6	26.6 ± 2.8	0.081
Diabetic retinopathy (n, %)	21 (16.0)	16 (18.0)	5 (14.0)	0.561
Diabetic nephropathy (n, %)	30 (20.1)	19 (16.8)	11 (29.7)	0.088
Fasting plasma glucose (mg/dl)	164.0 (136.0–202.0)	165.0 (134.0–202.5)	162.0 (137.0–198.5)	0.756
Baseline HbA1c (%(mmol/mol))	8.5 ± 1.3 (69.4 ± 13.7)	8.6 ± 1.3 (70.5 ± 14.3)	8.3 ± 1.1 (67.2 ± 12.4)	0.132
eGFR (mL/min/1.73 m^2^)	105.5 (89.9–114.4)	106.1 (89.9–115.2)	100.9 (90.4–112.4)	0.901
AST (IU/L)	27.4 ± 19.7	27.2 ± 21.7	27.8 ± 15.2	0.836
ALT (IU/L)	37.7 ± 27.1	38.5 ± 28.9	36.0 ± 23.3	0.566
Fasting C-peptide (ng/ml)	2.3 ± 1.7	2.3 ± 1.7	2.0 ± 1.4	0.371
Fasting Insulin (μU/ml)	8.8 ± 6.4	8.5 ± 6.2	9.5 ± 6.8	0.497
HOMA-IR	3.6 ± 2.7 2.7(1.8–4.4)	3.0 ± 1.9 2.6(1.8–4.1)	4.4 ± 3.3 3.3(1.7–5.9)	0.179
HOMA β-Cell function	29.1(13.9–46.4)	28.0 (13.6–47.2)	32.0 (19.8–46.0)	0.642
Previous treatment (n, %)				
Sulfonylureas	99 (53.8)	78 (62.9)	21 (35.0)	<0.001
Metformin	184 (100)	124 (100)	60 (100)	-
ACEi/ARB	74 (40.4)	50 (40.3)	24 (40.0)	0.967
Statins	115 (62.8)	80 (64.5)	35 (58.3)	0.417

Data are means ± SD, n (%), or median (interquartile range), or 95% CI. *p* < 0.05 was considered significant

DPP-4 dipeptidyl peptidase 4, SGLT2 sodium glucose cotransporter 2, BMI body mass index, eGFR estimated glomerular filtration rate, AST aspartate aminotransferase, ALT alanine transaminase, Hemoglobin A1c HbA1c, HOMA-IR homeostasis model assessment-insulin resistance, ACEi/ARB ACE inhibitor/angiotensin receptor blocker

The mean age of participants was 53.1 ± 6.9 years, the diabetic duration of the subjects was 7.1 ± 5.7 years, and mean BMI was 26.0 ± 3.4 kg/m^2^. In addition, 62.8% of patients had dyslipidemia and received statins. There were no differences in age, sex, duration of diabetes, and baseline laboratory findings including FPG, HbA1c, eGFR, aspartate aminotransferase, alanine transaminase, HOMA-IR, and HOMA- β-Cell function between the DPP-4 inhibitor group and the SGLT2 inhibitor group. There was a marginally significant difference in BMI between the DPP-4 inhibitor group and the SGLT2 inhibitor group. Besides, there was a significant difference in previous therapy with sulfonylureas because of South Korea’s health insurance coverage (62.9% vs. 35.0%, *p* < 0.001).

The 124 patients who received DPP-4 inhibitor included 55 patients with linagliptin and 69 patients with gemigliptin. The 55 patients with linagliptin did not have significant differences in baseline characteristics from the patients with gemigliptin with respect to age (54.0 ± 6.1 vs. 52.9 ± 7.8 years, *p* = 0.427), diabetic duration (7.3 ± 5.7 vs. 6.6 ± 5.6 years, *p* = 0.468), and BMI (26.2 ± 4.1 vs. 25.2 ± 3.1 years, *p* = 0.114) (Additional file 1: Table S1).

During the follow-up period, the mean FPG level in patients with DPP-4 inhibitor therapy including gemigliptin and linagliptin was reduced by 16.9 (95% CI, −30.5 to −9.5) mg/dl (*p* < 0.001). HbA1c was reduced by 0.8% (95% CI, −1.0 to −0.5) (*p* < 0.001). After 24 weeks of treatment with dapagliflozin, FPG was reduced by 24.8 (95% CI, −45.4 to −4.1) mg/dl (*p* = 0.020). HbA1c was reduced by 0.6% (95% CI, −0.9 to −0.3) (*p* < 0.001).

The difference of the change in the mean FPG (*p* = 0.462) and HbA1c (*p* = 0.593) between the DPP-4 inhibitor and the SGLT2 inhibitor was not significant in this study.

**Fig. 1:**
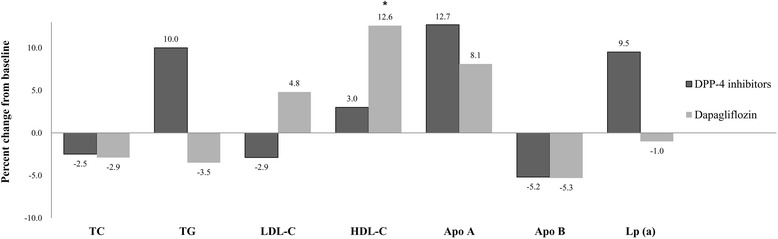
Changes in plasma lipid profile (%) by treatment of DPP-4 inhibitor and SGLT2 inhibitor. TC Total cholesterol, TG Triglycerides, HDL-C high-density lipoprotein cholesterol, LDL-C low-density lipoprotein cholesterol, Apo A apolipoprotein A, Apo B apolipoprotein B, Lp (a) lipoprotein (a). * *p* < 0.05 (comparison between DPP-4 inhibitor group and SGLT2 inhibitor group)

As shown in Table 2 and Fig. 1, there were different changes in the plasma lipid levels with treatment of the DPP-4 inhibitor and the SGLT2 inhibitor. After 24 weeks of treatment with the DPP-4 inhibitor including gemigliptin and linagliptin, TC was significantly reduced by 9.0 (95% CI, −15.8 to −2.1) mg/dl (*p* = 0.011), and LDL-C was reduced from 99.0 ± 33.9 mg/dl to 90.6 ± 28.6 (*p* = 0.004). The changes of TG (*p* = 0.011) and HDL-C (*p* = 0.426) were not significantly different.

**Table 2:** Effects of DPP-4 inhibitors and SGLT2 inhibitor on blood pressure, body weight, and glucose and lipid levels

	Total (*n =* 184)		DPP-4 inhibitors Linagliptin (*n =* 55) Gemigliptin (*n =* 69)		SGLT2 inhibitor (*n =* 60)		*p*-value
	Baseline	24 weeks	Baseline	24 weeks	Baseline	24 weeks	
Total cholesterol (mg/dl)	175.4 ± 39.6	167.0 ± 35.8*	174.9 ± 40.5	165.9 ± 34.4**	176.6 ± 38.2	169.2 ± 38.7	
Change from baseline	−8.5 (−13.7, −3.2)		−9.0 (−15.8, −2.1)		−7.4 (−15.4, 0.6)		0.836
Total triglycerides (mg/dl)	165.7 ± 96.2	150.5 ± 88.0*	172.9 ± 105.3	159.9 ± 94.5	150.3 ± 71.2	130.6 ± 68.6	
Change from baseline	−15.2 (−28.7, −1.3)		−13.0 (−30.8, 4.7)		−19.7 (−39.7, 0.3)		0.867
LDL cholesterol (mg/dl)	98.3 ± 32.7	93.0 ± 29.8*	99.0 ± 33.9	90.6 ± 28.6**	96.7 ± 30.3	97.9 ± 31.7	
Change from baseline	−5.3 (−9.6, −1.0)		−8.4 (−14.0, −2.8)		1.3 (−5.1, 7.6)		0.046***
HDL cholesterol (mg/dl)	43.5 ± 10.4	45.5 ± 11.0*	42.7 ± 10.2	42.2 ± 10.0	45.3 ± 10.6	50.3 ± 11.3**	
Change from baseline	2.0 (0.8, 3.2)		0.5 (−0.9, 2.0)		5.1 (3.0, 7.1)		0.001***
Apolipoprotein A (g/l)	121.6 ± 19.4	131.4 ± 26.8*	115.6 ± 18.5	120.5 ± 22.0	126.1 ± 17.9	139.8 ± 28.8**	
Change from baseline	9.8 (3.7, 16.0)		6.5 (−3.7, 12.6)		13.7 (4.8, 22.7)		0.726
Apolipoprotein B (g/l)	91.2 ± 21.5	86.4 ± 22.0	88.6 ± 21.8	81.3 ± 14.4	95.8 ± 21.3	90.3 ± 26.6	
Change from baseline	−4.8 (−11.3, 1.7)		−0.6 (−15.9, 8.3)		−5.5 (−13.5, 2.4)		0.660
Lipoprotein (a) (mg/dl)	17.7 ± 18.4	17.2 ± 19.8	17.2 ± 23.6	20.0 ± 25.4	15.6 ± 16.2	15.8 ± 19.3	
Change from baseline	−0.6 (−2.6, 1.5)		−0.9 (−3.4, 1.6)		0.2 (−4.2, 4.6)		0.991
Fasting plasma glucose (mg/dl)	172.8 ± 58.1	151.3 ± 44.9*	171.8 ± 58.8	150.8 ± 43.1**	174.8 ± 57.1	150.1 ± 48.7**	
Change from baseline	−21.5 (−31.2, −11.9)		−16.9 (−30.5, −9.5)		−24.8 (−45.4, −4.1)		0.462
HbA1c (%(mmol/mol))	8.5 ± 1.3 (69.4 ± 13.7)	7.8 ± 1.3* (61.9 ± 13.8)	8.6 ± 1.3 (70.5 ± 14.3)	7.9 ± 1.3** (62.6 ± 14.6)	8.3 ± 1.1 (67.2 ± 12.4)	7.7 ± 1.1** (60.3 ± 12.1)	
Change from baseline	−0.7 (−0.9, −0.5) −7.8 (−9.9, −5.7)		−0.8 (−1.0, −0.5) −8.2 (−10.9, −5.5)		−0.6 (−0.9, −0.3) −13.2 (−10.3, −3.5)		0.593
Systolic blood pressure (mmHg)	125.3 ± 12.9	124.5 ± 11.8	123.3 ± 14.1	124.4 ± 13.3	128.8 ± 10.1	124.6 ± 9.0**	
Change from baseline	−0.9 (−4.0, 2.3)		1.1 (−3.4, 5.7)		−4.2 (−7.6, −0.7)		0.112
Diastolic blood pressure (mmHg)	76.2 ± 12.2	74.5 ± 9.1	75.3 ± 12.3	74.7 ± 9.4	77.7 ± 12.1	74.3 ± 8.7**	
Change from baseline	−1.7 (−4.2, 0.8)		−0.6 (−3.9, 2.6)		−3.4 (−7.5, 0.7)		0.304
Body weight (kg)	69.6 ± 11.3	68.7 ± 11.1*	68.8 ± 10.9	68.3 ± 10.8	71.1 ± 11.9	69.5 ± 11.6**	
Change from baseline	−0.9 (−1.4, −0.4)		−0.5 (−0.7, −0.3)		−1.5 (−2.1, −0.9)		<0.001***

Data are means ± SD, or 95% CI. *p* < 0.05 was considered significant. Changes from baseline and percent change from baseline are adjusted for age, sex, diabetes duration, BMI, and glucose control status (HbA1c difference). DPP-4 dipeptidyl peptidase 4, SGLT2 sodium glucose cotransporter 2, LDL low-density lipoprotein, HDL high-density lipoprotein, Hemoglobin A1c HbA1c

* *p* < 0.05 (comparison between before treatment and after treatment in all subjects)

** *p* < 0.05 (comparison between before treatment and after treatment in each group)

*** *p* < 0.05 (comparison between DPP-4 inhibitor group and SGLT2 inhibitor group)

In patients who received 24 weeks of dapagliflozin, HDL-C was increased by 5.1 (95% CI, 3.0 to 7.1) mg/dl (*p* = 0.001). There were no significant differences in TC (*p* = 0.056), TG (*p* = 0.061), and LDL-C (*p* = 0.839). Apolipoprotein A was significantly increased by 13.7 ± 21.7 mg/dl (*p* = 0.005), whereas the change of apolipoprotein B was not significant (*p* = 0.505).

The change in the lipid profile between the DPP-4 inhibitor and dapagliflozin showed a significant difference in HDL-C (*p* = 0.001) and LDL-C (*p* = 0.046) after analysis from ANCOVA after adjustment of age, sex, diabetes duration, BMI, and change of HbA1c, after DPP-4 inhibitor or SGLT2 inhibitor therapy. The difference of the change in HDL-C was not caused by age (*p* = 0.300), sex (*p* = 0.173), or HbA1c (*p* = 0.314) difference or by BMI (*p* = 0.846). In addition, there was no significant difference of the change in HDL-C with respect to statin use (Additional file 1: Table S2 and Table S3).

In contrast with HDL-C, the difference of the change in LDL-C was associated with the change in HbA1c after DPP-4 inhibitor or SGLT2 inhibitor therapy (*p* = 0.032).

In addition, there was a significant reduction in the blood pressure and body weight from baseline to 24 weeks of SGLT2 inhibitor. The changes in systolic blood pressure, diastolic blood pressure, and body weight are presented in Table 2.

During the follow-up periods, among 55 patients, one patient had hypoglycemia with linagliptin and two patients had hypoglycemia and nausea with gemigliptin (Additional file 1: Table S4).

Among 60 patients, one patient with dapagliflozin had hypoglycemia, and two patients complained of having increased vaginal discharge and discomfort, but they recovered without medication (Additional file 1: Table S4).

## Discussion

Our observational study demonstrates that DPP-4 inhibitors and SGLT2 inhibitors have different effects on plasma lipid parameters in patients with type 2 diabetes. The DPP-4 inhibitor is associated with significant improvements in the TC and LDL-C levels, yet there were not significant differences in the TC level compared with the SGLT2 inhibitor. The SGLT2 inhibitor is associated with a significant increase in HDL-C, apolipoprotein A. Twenty-four weeks of SGLT2 inhibitor therapy shows a significant increase in HDL-C, LDL-C compared with the DPP-4 inhibitor.

The different effects on plasma lipid in this study are generally consistent with results from previous meta-analyses and randomized controlled trials [15, 18, 20, 22–34]. Sitagliptin has shown improvement on TG and HDL-C in patients with type 2 diabetes, but a meta-analysis found that the effect on lipid profile was not significant in patients with sitagliptin (*p* = 0.760) [12]. Vildagliptin was reported to have effects on TC and TG in patients with type 2 diabetes [19, 21]. Choe et al. reported that vildagliptin exerts a similar effect on glucose control, but exerts more effect on the lipid profile compared with sitagliptin [20]. Alogliptin has a beneficial effect on TC and TG, but results were variable across the studies [15, 18].

Several studies reported that linagliptin also has lipid-lowering effects [35]. Contrary to these reports, Owens et al. reported that linagliptin did not have significant effects on lipid profile after 24 weeks of linagliptin treatment compared with placebo as add-on metformin plus sulfonylurea therapy [17]. In a phase II trial, gemigliptin showed reduced TC and LDL-C at 12 weeks compared with the placebo, [16] although in a phase III trial, gemigliptin did not show a significant effect on the lipid profile at 24 weeks compared with the placebo [31].

The mechanism by which the DPP-4 inhibitor could influence the lipid profile in patients with type 2 diabetes has not been fully understood. This effect could be explained by glucagon-like peptide-1 receptor-mediated, DPP-4 inhibitor might have an inhibitory effect on lipid absorption in the gastrointestinal tract [36, 37].

The SGLT2 inhibitor inhibits glucose absorption and excretes glucose through urine and is related to calorie loss. Thus, the SGLT2 inhibitor induces switching from carbohydrate to lipid utilization for energy in the fasting state [22, 34]. It has been postulated that increased hepatic fatty acid levels may fuel the pool of acetyl-CoA, and induce both ketone body production and hepatic TC levels [23, 24, 34]. Empagliflozin was associated with a lowered LDL receptor expression and plasma LDL-C catabolism, which in turn increased LDL-C levels in an animal study [25, 34]. Canagliflozin has been related with a mean-percentage increase of LDL-C of 4.5% and 8.0% for 100 mg and 300 mg, respectively, compared with placebo. In addition, significant increases in HDL-C were observed with treatment of canagliflozin compared with placebo in four of eight phase III trials [26]. One previous report stated that the mean percent changes from baseline at 24 weeks were −1.0% vs. 2.9% for LDL-C in the placebo and dapagliflozin 10 mg groups, respectively [38].

Dyslipidemia is associated with an increased risk of CVD in subjects with type 2 diabetes [28]. In this study, the change in mean HDL-C from baseline to 24 weeks of DPP-4 inhibitor or SGLT2 inhibitor therapy showed a significant difference. Although both agents raised HDL-C, the SGLT2 inhibitor raised more HDL-C. Additionally, this study showed consistent different effects on HDL-C between DPP-4 inhibitor and SGLT2 inhibitor after analyzing the subjects receiving statins and those not receiving statins separately. Low HDL-C has been reported to be an independent risk factor for CVD in patients with type 2 diabetes [29]. In the Framingham Heart Study, an increased risk of myocardial infarction was reported to be approximately 25% for every 5 mg/dl decrease in serum HDL-C [30]. Ogita et al. reported that low HDL-C is a residual risk factor for cardiovascular outcome despite optimal LDL-C in patients with type 2 diabetes with stable coronary artery disease [39]. This study suggests that dapagliflozin may be preferred in patients with low HDL-C.

In addition, this study found that the DPP-4 inhibitor and the SGLT2 inhibitor differed with opposite effects on LDL-C. Whereas the SGLT2 inhibitor is reported to be related with an increased LDL-C, this study did not show a significant difference in LDL-C after 24 weeks of dapagliflozin treatment in patients with type 2 diabetes. One recent study reported that empagliflozin was associated with a lower rate of cardiovascular outcome [40]. The clinical implication of the SGLT2 inhibitor and dyslipidemia for CVD needs to be analyzed in further studies.

There were several limitations to this study. First, our study was conducted in a single center and the sample size was relatively small. Second, this study is a retrospective study; thus, the medications were not controlled, and the participants received metformin and/or a sulfonylurea with varying doses. However, we did not permit changes of medication, including anti-diabetic agent, statin, fenofibrate, and any other medication, during the follow-up periods. Third, we excluded the patients who had changed medication during the follow-up periods, and the side effects of the drugs may be underestimated in this study. Fourth, this study was composed of only the Korean population. Further studies are needed to apply the result of this study to other populations.

## Conclusions

In conclusion, a DPP-4 inhibitor and an SGLT2 inhibitor, when added to metformin and/or a sulfonylurea, have a modest beneficial effect in glucose control and have different effects in lipid profile in patients with type 2 diabetes. Either a DPP-4 inhibitor or an SGLT2 inhibitor may be beneficial in patients with type 2 diabetes for CVD. There were significant differences in the change of HDL-C, LDL-C between DPP-4 inhibitor and SGLT2 inhibitor therapy. Thus, an SGLT2 inhibitor may be preferred as an add-on to metformin and/or a sulfonylurea in patients with low HDL-C.

## Additional file

Additional file 1: Table S1. Comparison of baseline characteristics in subjects with linagliptin and gemigliptin. **Table S2.** Effects of DPP-4 inhibitors and SGLT2 inhibitor on lipid levels in subjects receiving statins. **Table S3.** Effects of DPP-4 inhibitors and SGLT2 inhibitor on lipid levels in subjects not receiving statins. **Table S4.** Summary of adverse events after 24 weeks of DPP-4 inhibitor of SGLT2 inhibitor. (DOCX 36 kb)

## Abbreviations

ADA: American diabetes association
ANCOVA: Analysis of covariance
BMI: Body mass index
CVD: Cardiovascular disease
DPP-4: Dipeptidyl peptidase-4
eGFR: Estimated glomerular filtration rate
FPG: Fasting plasma glucose
HbA1c: Hemoglobin A1c
HDL-C: HDL-cholesterol
HOMA-IR: Homeostatic model assessment-insulin resistance
LDL-C: LDL-cholesterol
Lp(a): Lipoprotein (a)
SGLT2: Sodium glucose cotransporter 2
TC: Total cholesterol
TG: Triglyceride
