# Supplementary material for: A comparison of effects of DPP-4 inhibitor and SGLT2 inhibitor on lipid profile in patients with type 2 diabetes
Source: Lipids Health Dis. 2017 Apr 13;16:58. doi: 10.1186/s12944-017-0443-4 (PMC5390350; doi:10.1186/s12944-017-0443-4)
Supplement: Additional file 1: Table S1. — Comparison of baseline characteristics in subjects with linagliptin and gemigliptin. Table S2. Effects of DPP-4 inhibitors and SGLT2 inhibitor on lipid levels in subjects receiving statins. Table S3. Effects of DPP-4 inhibitors and SGLT2 inhibitor on lipid levels in subjects not receiving statins. Table S4. Summary of adverse events after 24 weeks of DPP-4 inhibitor of SGLT2 inhibitor. (DOCX 36 kb) [file 12944_2017_443_MOESM1_ESM.docx]

**Table S1. Comparison of baseline characteristics in subjects with linagliptin and gemigliptin.**

|  | Total | Linaglipitin | Gemigliptin | *p*-value |
| --- | --- | --- | --- | --- |
| n | 124 | 55 | 69 |  |
| Women (n, %) | 62 (50.0) | 23 (41.8) | 39 (56.5) | 0.104 |
| Age (years) | 53.4 ± 7.1 | 54.0 ± 6.1 | 52.9 ± 7.8 | 0.427 |
| Duration of diabetes (years) | 6.9 ± 5.6 | 7.3 ± 5.7 | 6.6 ± 5.6 | 0.468 |
| Hypertension (n, %) | 47 (37.9) | 21 (38.2) | 26 (37.7) | 0.954 |
| Smoking (n, %) |  |  |  | 0.313 |
| Current (n, %) | 30 (25.9) | 17 (32.7) | 13 (20.3) |  |
| Ex-smoker (n, %) | 13 (11.2) | 5 (9.6) | 8 (12.5) |  |
| Body weight (kg) | 68.8 ± 10.9 | 70.7 ± 12.0 | 67.4 ± 9.9 | 0.096 |
| BMI (kg/m^2^) | 25.6 ± 3.6 | 26.2 ± 4.1 | 25.2 ± 3.1 | 0.114 |
| Fasting plasma glucose (mg/dl) | 171.8 ± 58.8 | 171.5 ± 48.9 | 172.1 ± 65.8 | 0.962 |
| Baseline HbA1c (%(mmol/mol)) | 8.6 ± 1.3  (70.5 ± 14.3) | 8.7 ± 1.4  (71.4 ± 15.8) | 8.5 ± 1.2  (69.7 ± 13.0) | 0.528 |
| eGFR (mL/min/1.73m^2^) | 105.8 ± 26.6 | 103.0 ± 24.9 | 108.1 ± 27.8 | 0.285 |
| Fasting C-peptide (ng/ml) | 2.3 ± 1.7 | 2.2 ± 1.3 | 2.4 ± 1.9 | 0.650 |
| Fasting Insulin (µU/ml) | 8.5 ± 6.2 | 9.1 ± 5.3 | 8.2 ± 6.7 | 0.548 |
| Previous treatment (n, %) | |  |  |  |
| Sulfonylureas | 78 (62.9) | 34 (61.8) | 44 (63.8) | 0.823 |
| Metformin | 124 (100) | 55 (100) | 69 (100) | - |
| ACEi/ARB | 50 (40.3) | 25 (45.5) | 25 (36.2) | 0.298 |
| Statins | 80 (64.5) | 37 (67.3) | 43 (62.3) | 0.567 |

Data are means ± SD, n (%). *p* < 0.05 was considered significant.

BMI body mass index, eGFR estimated glomerular filtration rate, ACEi/ARB ACE inhibitor/angiotensin receptor blocker.

**Table S2. Effects of DPP-4 inhibitors and SGLT2 inhibitor on lipid levels in subjects receiving statins.**

|  | Total  (n=115) | | DPP-4 inhibitors  (n=80) | | SGLT2 inhibitor  (n=35) | | *p*-value |
| --- | --- | --- | --- | --- | --- | --- | --- |
|  | Baseline | 24 weeks | Baseline | 24 weeks | Baseline | 24 weeks |  |
| Total cholesterol (mg/dl) | 167.2 ± 39.8 | 159.0 ± 30.8* | 166.5 ± 41.2 | 158.0 ± 31.2 | 168.6 ± 36.8 | 161.2 ± 30.2 |  |
| Change from baseline | -8.2 (-15.2, -1.1) | | -8.5 (-17.4, -0.4) | | -7.4 (-19.2, 4.4) | | 0.830 |
| Total triglycerides (mg/dl) | 173.9 ± 86.0 | 155.3 ± 80.5* | 181.2 ± 93.6 | 161.5 ± 82.7** | 156.6 ± 62.5 | 140.7 ± 74.2 |  |
| Change from baseline | -18.5 (-33.3, 3.7) | | -19.7 (-38.0, 1.3) | | -15.8 (-41.7, 9.9) | | 0.758 |
| LDL cholesterol (mg/dl) | 90.3 ± 29.2 | 84.3 ± 25.3* | 90.9 ± 30.4 | 82.3 ± 25.6****** | 89.1 ± 26.3 | 89.0 ± 24.2 |  |
| Change from baseline | -6.0 (-11.7, -0.4) | | -8.5 (-15.6, -1.5) | | -0.1 (-9.5, 9.2) | | 0.196 |
| HDL cholesterol (mg/dl) | 42.9 ± 9.4 | 45.2 ± 10.6* | 42.0 ± 9.2 | 43.1 ± 10.4 | 45.0 ± 9.6 | 50.2 ± 9.4****** |  |
| Change from baseline | 2.3 (0.8, 3.8) | | 1.0 (-0.7, 2.8) | | 5.2 (2.7, 7.2) | | 0.005*** |
| Apolipoprotein A (g/l) | 120.3 ± 18.4 | 129.5 ± 25.2* | 112.7 ± 17.3 | 118.7 ± 19.3 | 128.0 ± 16.6 | 140.4 ± 24.5****** |  |
| Change from baseline | 9.2 (2.0, 16.4) | | 6.0 (-4.4, 16.4) | | 12.4 (1.1, 23.7) | | 0.812 |
| Apolipoprotein B (g/l) | 83.5 ± 19.1 | 78.9 ± 14.0 | 79.6 ± 21.2 | 78.3 ± 12.9 | 87.4 ± 16.7 | 79.5 ± 15.5 |  |
| Change from baseline | -4.6 (-14.1, 5.0) | | -1.3 (-18.2, 15.5) | | -7.8 (-19.4, 3.7) | | 0.280 |
| Lipoprotein (a) (mg/dl) | 18.6 ± 20.5 | 19.0 ± 23.2 | 17.4 ± 20.3 | 17.3 ± 22.5 | 25.5 ± 24.8 | 29.4 ± 30.0 |  |
| Change from baseline | 0.5 (-1.9, 2.8) | | -0.1 (-2.7, 2.4) | | 4.0 (-9.5, 17.3) | | 0.556 |

Data are means ± SD, or 95 % CI. *p* < 0.05 was considered significant. Changes from baseline and percent change from baseline are adjusted for age, sex, diabetes duration, BMI, and glucose control status (HbA1c difference), . DPP-4 dipeptidyl peptidase 4, SGLT2 sodium glucose cotransporter 2, LDL low-density lipoprotein, HDL high-density lipoprotein.

* *p* < 0.05 (comparison between before treatment and after treatment in all subjects)

** *p* < 0.05 (comparison between before treatment and after treatment in each group)

*** *p* < 0.05 (comparison between DPP-4 inhibitor group and SGLT2 inhibitor group)

**Table S3. Effects of DPP-4 inhibitors and SGLT2 inhibitor on lipid levels in subjects not receiving statins.**

|  | Total  (n=69) | | DPP-4 inhibitors  (n=44) | | SGLT2 inhibitor  (n=25) | | *p*-value |
| --- | --- | --- | --- | --- | --- | --- | --- |
|  | Baseline | 24 weeks | Baseline | 24 weeks | Baseline | 24 weeks |  |
| Total cholesterol (mg/dl) | 189.2 ± 35.7 | 180.3 ± 39.6* | 190.2 ± 40.5 | 180.3 ± 34.4 | 187.6 ± 38.1 | 180.3 ± 46.6 |  |
| Change from baseline | -9.0 (-16.8, -1.1) | | -9.9 (-20.9, 1.1) | | -7.4 (-18.0, 3.3) | | 0.915 |
| Total triglycerides (mg/dl) | 152.0 ± 110.5 | 142.5 ± 99.4 | 157.8 ± 123.6 | 156.9 ± 113.9 | 141.3 ± 82.6 | 116.2 ± 58.4 |  |
| Change from baseline | -9.5 (-36.6, 17.6) | | -1.0 (-39.2, 37.3) | | -25.2 (-59.1, 8.7) | | 0.557 |
| LDL cholesterol (mg/dl) | 111.4 ± 34.2 | 107.3 ± 31.3 | 113.9 ± 35.1 | 105.7 ± 28.0 | 106.9 ± 32.8 | 110.1 ± 36.9 |  |
| Change from baseline | -4.1 (-11.0, 2.8) | | -8.2 (-17.9, 1.5) | | 3.2 (-5.5, 11.9) | | 0.154 |
| HDL cholesterol (mg/dl) | 44.5 ± 11.9 | 46.0 ± 11.6 | 43.8 ± 11.9 | 43.5 ± 9.5 | 45.6 ± 12.1 | 50.4 ± 13.7****** |  |
| Change from baseline | 1.5 (-0.7, 3.7) | | 0.3 (-3.1, 2.4) | | 4.8 (1.2, 8.4) | | 0.038*** |
| Apolipoprotein A (g/l) | 123.4 ± 21.2 | 134.2 ± 29.5 | 122.0 ± 27.0 | 122.4 ± 19.4 | 124.0 ± 19.8 | 139.2 ± 32.3 |  |
| Change from baseline | 10.8 (-1.2, 22.8) | | 0.4 (-18.6, 19.4) | | 15.2 (-0.9, 31.2) | | 0.047*** |
| Apolipoprotein B (g/l) | 103.0 ± 21.5 | 97.9 ± 22.0 | 98.2 ± 11.6 | 88.0 ± 4.3 | 105.0 ± 22.6 | 102.0 ± 30.6 |  |
| Change from baseline | -5.1 (-14.1, 3.9) | | -10.2 (-23.0, 2.6) | | -3.0 (-15.6, 9.6) | | 0.180 |
| Lipoprotein (a) (mg/dl) | 16.2 ± 14.5 | 13.7 ± 10.8 | 22.8 ± 17.8 | 19.2 ± 11.6 | 16.7 ± 9.1 | 9.1 ± 8.4 |  |
| Change from baseline | -2.5 (-6.7, 1.7) | | -3.6 (-13.2, 6.0) | | -1.6 (-7.2, 4.0) | | 0.974 |

Data are means ± SD, or 95 % CI. *p* < 0.05 was considered significant. Changes from baseline and percent change from baseline are adjusted for age, sex, diabetes duration, BMI, and glucose control status (HbA1c difference). DPP-4 dipeptidyl peptidase 4, SGLT2 sodium glucose cotransporter 2, LDL low-density lipoprotein, HDL high-density lipoprotein.

* *p* < 0.05 (comparison between before treatment and after treatment in all subjects)

** *p* < 0.05 (comparison between before treatment and after treatment in each group)

*** *p* < 0.05 (comparison between DPP-4 inhibitor group and SGLT2 inhibitor group)

**Table S4. Summary of adverse events after 24 weeks of DPP-4 inhibitor of SGLT2 inhibitor.**

|  | Number of subjects | | | Incidence (%) | |
| --- | --- | --- | --- | --- | --- |
|  | DPP-4 inhibitors  (n = 124) | SGLT2 inhibitor  (n = 60) | DPP-4 inhibitors  (n = 124) | | SGLT2 inhibitor  (n = 60) |
| All adverse events | 4 | 3 | 3.2 | | 5 |
| Main adverse events |  | | | | |
| Vulvovaginal candidiasis | 0 | 2 | 0 | | 3.3 |
| Nausea | 1 | 0 | 0.8 | | 0 |
| Hypoglycemia | 3 | 1 | 2.4 | | 1.7 |

Data are n or (%).

DPP-4 dipeptidyl peptidase 4, SGLT2 sodium glucose cotransporter 2.
